# External Validation of SAFE Score to Predict Atrial Fibrillation Diagnosis after Ischemic Stroke: A Retrospective Multicenter Study

**DOI:** 10.1155/2023/6655772

**Published:** 2023-12-07

**Authors:** Miguel Quesada López, Laura Amaya Pascasio, Sara Blanco Madera, Jorge Pagola, Diana Vidal de Francisco, Elena de Celis Ruiz, Inmaculada Villegas Rodríguez, Joaquín Carneado-Ruiz, Juan Antonio García-Carmona, Juan Manuel García Torrecillas, Ana López Ferreiro, Iker Elosua Bayes, Ricardo Jaime Rigual Bobillo, María Isabel López López, Íñigo Esain González, María Dolores Ortega Ortega, Marina Blanco Ruiz, Irene Pérez Ortega, Carlos Lázaro Hernández, Blanca Fuentes Gimeno, Antonio Arjona Padillo, Patricia Martínez Sánchez

**Affiliations:** ^1^Neurology, Hospital Universitario Torrecárdenas, Almería, Spain; ^2^Neurology, Hospital Universitario Virgen de las Nieves, Granada, Spain; ^3^Neurology, Hospital Universitari Vall d'Hebron, Barcelona, Spain; ^4^Neurology, Hospital Universitario de León, León, Spain; ^5^Neurology, Hospital La Paz Institute for Health Research-IdiPaz (La Paz University Hospital-Universidad Autónoma de Madrid, Spain; ^6^Neurology, Hospital Universitario San Cecilio, Granada, Spain; ^7^Neurology, Hospital Universitario Puerta de Hierro, Madrid, Spain; ^8^Neurology, Hospital Universitario Santa Lucía, Cartagena, Spain; ^9^Emergency and Research Unit, Torrecárdenas University Hospital, 04009 Almería, Spain; ^10^CIBER de Epidemiología y Salud Pública (CIBERESP), 28029 Madrid, Spain; ^11^Instituto de Investigación Biosanitaria Ibs, 18012 Granada, Spain; ^12^Faculty of Health Science, Health Research Center (CEINSA), University of Almería, Spain

## Abstract

**Introduction:**

The screening for atrial fibrillation (AF) scale (SAFE score) was recently developed to provide a prediction of the diagnosis of AF after an ischemic stroke. It includes 7 items: *age* ≥ 65 years, bronchopathy, thyroid disease, cortical location of stroke, intracranial large vessel occlusion, NT-ProBNP ≥250 pg/mL, and left atrial enlargement. In the internal validation, a good performance was obtained, with an *AUC* = 0.88 (95% CI 0.84-0.91) and sensitivity and specificity of 83% and 80%, respectively, for *scores* ≥ 5. The aim of this study is the external validation of the SAFE score in a multicenter cohort.

**Methods:**

A retrospective multicenter study, including consecutive patients with ischemic stroke or transient ischemic attack between 2020 and 2022 with at least 24 hours of cardiac monitoring. Patients with previous AF or AF diagnosed on admission ECG were excluded.

**Results:**

Overall, 395 patients were recruited for analysis. The SAFE score obtained an *AUC* = 0.822 (95% CI 0.778-0.866) with a sensitivity of 87.2%, a specificity of 65.4%, a positive predictive value of 44.1%, and a negative predictive value of 94.3% for a SAFE *score* ≥ 5, with no significant gender differences. Calibration analysis in the external cohort showed an absence of significant differences between the observed values and those predicted by the model (Hosmer-Lemeshow's test 0.089).

**Conclusions:**

The SAFE score showed adequate discriminative ability and calibration, so its external validation is justified. Further validations in other external cohorts or specific subpopulations of stroke patients might be required.

## 1. Introduction

Despite an extensive etiological study, 26-40% of ischemic strokes remain without a well-defined cause [1–3], so they are called cryptogenic strokes (CS). Occult atrial fibrillation (AF), which is often paroxysmal and asymptomatic, is an important underlying cause of CS [4] and, if detected, the risk of stroke recurrence would be drastically reduced with anticoagulant treatment [5]. Moreover, AF-related strokes are generally more extensive, causing higher health costs, higher lethality, and disability, as well as a higher recurrence rate [6].

The diagnosis of occult AF is currently a challenge, with different diagnostic devices available, from external recorders to subcutaneous insertable cardiac monitors (ICMs). In patients with CS, prolonged ECG monitoring (up to 36 months) with ICMs shows an incidence of occult AF of 30%, much higher than with conventional devices [7]. However, the number of patients who benefit from them is small due to the invasive procedure and the limited availability of technical, human, and economic resources. Patient selection based on well-known AF-risk factors may improve diagnostic yield and opportunities for treatment and prevention of stroke recurrence [8]. Recently, an ESO guideline on screening for subclinical AF after stroke or transient ischemic attack (TIA) of undetermined origin [9] strongly recommends extending cardiac monitoring time to more than 48 hours, although with low certainty of the evidence of the upper monitoring limit. In addition, it finds weak evidence at the moment to use biomarkers predictive of AF to identify patients at higher risk, recognizing this area as important for future research.

In this context, it is necessary to optimize tools that allow clinicians to predict which ischemic stroke/TIA patients are more likely to have occult AF. A predictive model of AF (property registration number 2106148086772) has been developed in a single-center retrospective cohort of patients with ischemic stroke treated at a tertiary stroke center, the screening for AF scale (SAFE score) [10]. This score combines the majority of parameters that have been described as being associated with AF (clinical, echocardiographic, analytical, and neuroimaging) and includes age, bronchopathy, thyroid disease, N-terminal pro-B-type natriuretic peptide (NT-ProBNP), left atrial enlargement (LAE), cortical topography of stroke, and intracranial large vessel occlusion (LVO) (Table 1). The score obtained a good performance in the validation cohort, with an AUC (area under the ROC curve) of 0.88 (95% confidence interval 0.84–0.91). *Punctuation* ≥ 5 was related to patients with paroxysmal AF with a sensitivity of 83%, a specificity of 80%, and a negative predicted value of 94%.

The present study is aimed at carrying out the external validation of the SAFE score in an independent multicenter cohort of patients, including a gender perspective.

## 2. Methods

### 2.1. Patient Selection

This multicenter retrospective study was conducted across eight comprehensive stroke centers in Spain. Regarding inclusion criteria, consecutive patients over 18 years of age who were admitted for ischemic stroke or TIA between January 2020 and February 2022 were selected. Patients from Torrecardenas University Hospital included in the internal validation cohort (those between January 2020 and May 2021) were excluded from this external validation cohort. Moreover, the enrollment period for each center was determined according to the availability of the necessary complementary studies to fulfill the inclusion criteria of the study.

All included patients underwent an etiological assessment, which involved an evaluation of cerebral arteries by angio-CT or neurosonological study, a minimum cardiac monitoring of 24 hours, a determination of NT-ProBNP levels during the admission, and an echocardiography/focused cardiac ultrasound either during admission or within the first 6 months after the index event.

Patients with known AF or those diagnosed with AF on the initial electrocardiogram (ECG), patients with other major cardioembolic sources (severe ventricular dysfunction, mechanical prosthetic valve, and rheumatic mitral stenosis), and patients with hemorrhagic strokes were excluded.

### 2.2. Data Collection and Variable Definition

The database of the study has been designed on the REDCap (Research Electronic Data Capture) platform [11, 12], an encrypted web platform for managing databases.

A diagnosis of AF was considered during admission or within 12 months after the ischemic event, with a tracing of at least 30 seconds of a heart rhythm without P waves and irregular RR intervals. The diagnosis of AF could have been made by the treating neurologist, by a cardiologist, or by any physician attending to the patient during the period under consideration.

The SAFE scale variables were defined as follows: [10] (a) age was scored when it was greater than or equal to 65 years; (b) for the diagnosis of bronchopathy, comorbidity with chronic obstructive pulmonary disease (COPD), obstructive sleep apnea (OSA), or bronchial hyperresponsiveness without meeting the criteria for COPD has been considered; (c) thyroid disease was noted when the patient presented with hyper- or hypothyroidism, excluding subclinical forms; (d) NT-ProBNP was scored when it was greater than or equal to 250 pg/mL (limits established in the internal validation); (e) LAE was defined as atrial enlargement of any degree, following the recommendations of the American Society of Echocardiography and the European Association of Cardiovascular Imaging; [13] (f) the cortical topography of stroke was defined as the nonlacunar involvement of the cerebral or cerebellar hemispheres on neuroimaging; and finally, (g) intracranial LVO was defined as the occlusion of a large vessel responsible for the patient's symptom, excluding isolated occlusions of extracranial arteries.

### 2.3. Statistical Analysis

Data analysis was performed using SPSS software v26 (IBM Inc., Armonk, NY, USA) and R Statistical Software v4.1.2 (R Core Team 2021). For the descriptive study, summary measures were obtained for quantitative (medians and interquartile range) and qualitative (percentages and frequency distributions) variables. Comparisons between qualitative variables were performed using Pearson's *X*^2^ or Fisher's exact test when appropriate; comparisons between quantitative variables were performed using Student's test for independent data or the Mann–Whitney test when appropriate. Similarly, a univariate analysis was performed based on gender. The performance of the score and the discriminative ability of the predictive model were calculated for each gender by comparing the area under the curve (AUC) for each subgroup.

For this report, the model was retested on the internal validation cohort using the repeated cross-validation method with *k* = 5 and 10 repetitions. Subsequently, the model was trained on the external multicenter cohort, obtaining in both cases the corresponding confusion matrix. For external validation, the performance of the initial model was compared with that of the external cohort. In addition, calibration was evaluated using the Hosmer-Lemeshow test, taking into account both its level of significance and its graphic representation.

## 3. Results

**Figure 1:**
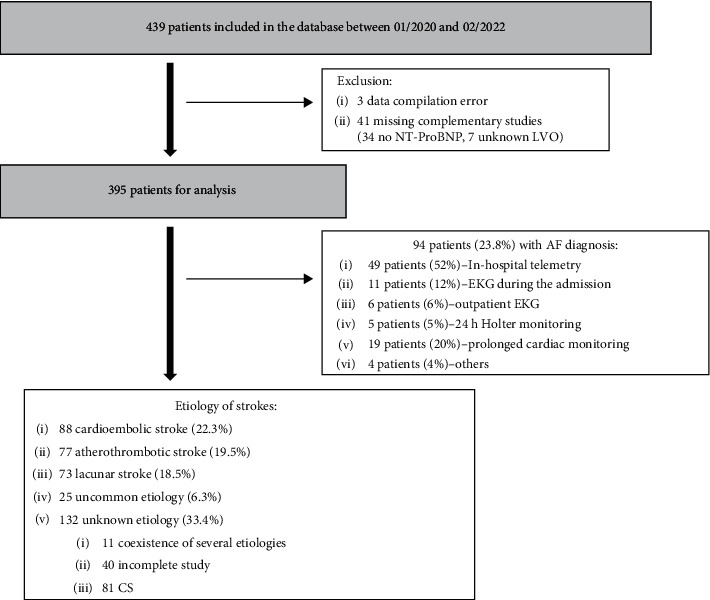
Flow diagram of study participants. CS: cryptogenic stroke; EKG: electrocardiogram; LVO: large vessel occlusion; NT-ProBNP: N-terminal pro-B-type natriuretic peptide.

From a total of 439 patients included in the database, 395 were finally selected for the analysis (Figure 1). Patient characteristics, laboratory data, echocardiographic, and neuroimaging features are detailed in Table 2. These characteristics have also been assessed according to gender in Table S1 of the Supplementary material file. Considering the data obtained in the internal validation study [10], the main difference was a greater age in our population (patients with *age* ≥ 65 years, 68.1% versus 50.4% in the internal validation study). Of all the variables included in the SAFE score, only thyroid disease did not show a significant difference in this external cohort.

In terms of performance, a SAFE score of ≥5 points achieved a sensitivity of 87.2%, a specificity of 65.4%, a positive predictive value (PPV) of 44.1%, and a negative predictive value (NPV) of 94.3% for AF diagnosis. These parameters were similar between genders for the abovementioned cut-off point (sensitivity males 83%, females 91%; specificity males 66%, females 65%; PPV males 38%, females 52%; NPV males 94%, females 95%).

**Figure 2:**
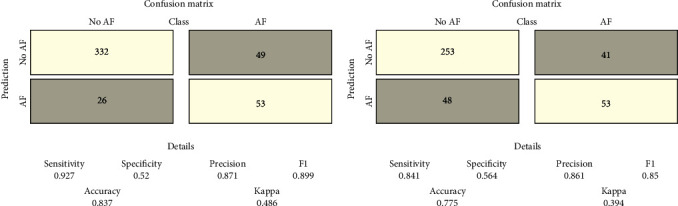
Repeated cross-validation method with *k* = 5 and 10 repetitions. (a) Confusion matrix of the model retested on the internal validation cohort. (b) Confusion matrix of the model trained on the external multicenter cohort. AF: atrial fibrillation diagnosis; no AF: absence of atrial fibrillation diagnosis.

When the model was retested in the internal validation cohort using the repeated cross-validation method, the confusion matrix reported high values for sensitivity (0.927), precision (0.871), and accuracy (0.837) (Figure 2(a)). Further, the model was trained on the external multicenter cohort, achieving values in the confusion matrix that were again remarkable in terms of sensitivity (0.841), precision (0.861), and accuracy (0.775) (Figure 2(b)).

**Figure 3:**
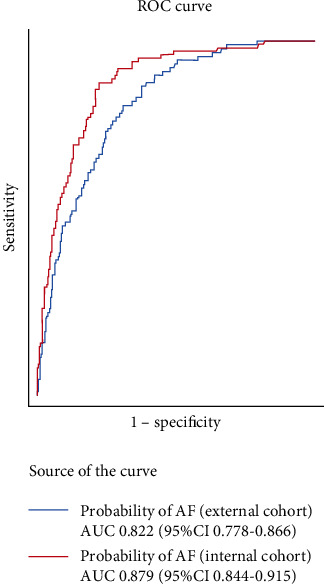
Receiver operating characteristic (ROC) curve showing the atrial fibrillation (AF) prediction by SAFE in the internal and external cohorts.

**Figure 4:**
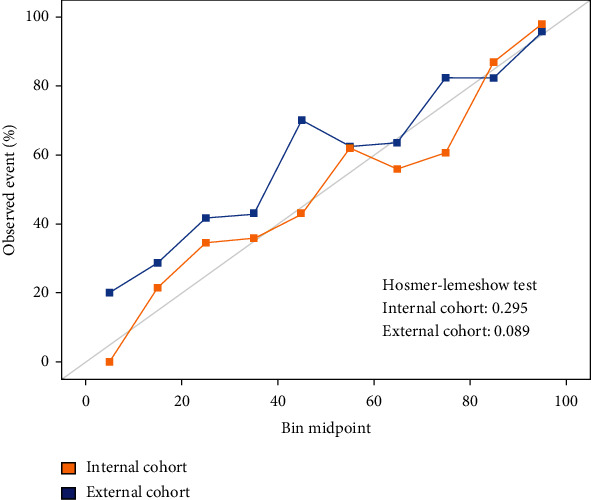
Graphic representation of the Hosmer-Lemeshow test showing nonsignificant differences between observed and predicted values in both cohorts.

The discriminative ability, measured by the AUC, reported a value of 0.879 (95% CI 0.844-0.915) in the internal cohort versus 0.822 (95% CI 0.778-0.866) in the external cohort (Figure 3). The AUC in this external cohort did not differ significantly between genders (Figure S1, Supplementary material file). Concerning the calibration of the initial model (internal cohort) and that applied to the external cohort, in both cases, a nonsignificant result was obtained in the Hosmer-Lemeshow test (0.295 and 0.089, respectively) (Figure 4).

## 4. Discussion

This retrospective multicenter study provides evidence that the SAFE score is a valid tool for predicting the risk of hidden AF in ischemic stroke patients. The model is highlighted for its sensitivity and precision, as well as a very high NPV for a cutoff point of ≥5 points. Thus, it could be useful for optimizing resources for the diagnosis of occult AF in these patients.

Left atrial cardiomyopathy (LACM) was defined in 2016 as “any complex of structural, architectural, contractile, or electrophysiological changes affecting the atria with the potential to produce clinically relevant manifestations” [14]. A bidirectional relationship between LACM and AF is postulated, assuming in many cases that LACM precedes the onset of AF, and conversely, AF may act as a trigger for atrial remodeling [15]. Although the diagnostic criteria are not completely defined, factors such as NT-ProBNP, obstructive sleep apnea, or LAE are included. On the other hand, “lone AF” is described when no apparent explanation or underlying comorbidity can be identified, with a very low embolic risk (only a 1-2% cumulative 15-year risk of stroke) [16]. However, with aging or the concurrence of vascular risk factors, the embolic risk associated with this “lone AF” increases [14]. These facts emphasize the great importance of having a reliable risk scale that includes factors associated with atrial disease, such as those contained in the SAFE score.

Different scores described in the literature can be found with the same purpose as the present one [17], but only two of them reflect all the types of variables included in the SAFE score. The model published by Seo et al. [18] reports clinical (age), echocardiographic (left atrial size), laboratory (free fatty acid and triglycerides), and neuroimaging (susceptibility vessel sign, hemorrhagic transformation, and cortical stroke involvement) variables. It is noteworthy that no brain natriuretic peptide is included in the score since it is the analytical parameter most closely related to AF [19]. Furthermore, it has not been externally validated in an independent cohort in addition to the one initially published. In the internal validation, the performance of this model was described with a C-index of 0.908 (95% CI 0.887-0.930) [18]. Kneihsl et al. have recently published the Graz AF risk score [20], which also includes clinical (age), echocardiographic (left ventricular ejection fraction, LAE), laboratory (NT-ProBNP), neuroimaging (recurrent stroke under antiplatelet treatment, multiterritory brain infarct, and prior cortical/cerebellar infarction) factors, and electrocardiographic variables (supraventricular premature beats or atrial *runs* > 20 beats). This scale was not developed in a cohort of stroke patients but was based on a bibliographic search by the authors. The Graz AF risk score was validated in a single-center prospective cohort of CS, involving only 24 patients with AF, and achieved an AUC of 0.85 (95% CI 0.78-0.92).

Not all the scales published in the literature report external validations. In a recent systematic review [17], twelve of the seventeen scores included were externally validated. The STAF score [21] is probably the one that accumulates the most external validations, being a scale that incorporates four parameters (age, baseline NIHSS score, LAE, and absence of established vascular etiology). Although they have positive validations [22, 23], some of them show limited utility [24, 25] or show a reduced yield in a cohort of patients with CS [26]. Indeed, it is common to find a worse performance of the scales in external cohorts other than those of the original validation. The same applies to the HAVOC score [27], a scale with 7 items (age, hypertension, valve disease, peripheral vascular disease, obesity, congestive heart failure, and coronary artery disease), which also performed worse in a cohort of embolic strokes of undetermined source (ESUS) [28]. The same effect was found in the study by Chen et al. [29] with LADS [30] and iPAB [31] scores or in the study by Kneihsl et al. [20] with the CHADS2 [32] and AS5F [33] scores.

There are several limitations related to this study. First is the retrospective design with a moderate number of patients. Secondly, there could be significant heterogeneity in the etiological study performed in the different participant centers. Finally, other than the minimum required 24 h cardiac monitoring, only 23% of patients had an additional 24 h Holter and another 17.7% had a 28-day Holter (Table 2). This unavailability of prolonged cardiac monitoring in all patients may have resulted in the misdiagnosis of some cases of AF.

## 5. Conclusion

The SAFE score showed adequate discriminative ability in the external cohort assessed by AUC, with no significant differences between the values obtained and those predicted in terms of calibration, so its external validation is justified. This score could be a useful tool for the identification of patients with ischemic stroke at high risk of occult AF, thus allowing to optimize the resources needed for its diagnosis. Further validations of the SAFE score in other external cohorts or specific subpopulations of stroke patients, such as CS or ESUS, as well as with prolonged cardiac monitoring, might be required.

## Figures and Tables

**Table 1:** SAFE score.

Variable	Points
Age	
<65 years	0
≥65 years	2
COPD or OSA	1
Thyroid disease	1
NT-ProBNP	
<250 pg/mL	0
≥250 pg/mL	2
Left atrial enlargement	2
Cortical topography of stroke	1
Intracranial large vessel occlusion	1
Total	0 to 10

COPD: chronic obstructive pulmonary disease; NT-ProBNP: N-terminal pro-B-type natriuretic peptide; OSA: obstructive sleep apnea.

**Table 2:** Patient characteristics (between brackets, data of the internal validation cohort [11]).

Variable	All patients ^∗^ (*n* = 395)	Atrial fibrillation ^∗^ (*n* = 94)	Nonatrial fibrillation ^∗^ (*n* = 301)	*P* value ^∗∗^
*Clinical variables*				
Median age (IQR), years	72 (20) [65]	77.5 (16) [74]	69 (22) [62]	**<0.001**
*Age* ≥ 65 years, *n* (%)	269 (68.1%) [50.4%]	87 (92.6%) [86.3%]	182 (60.5%) [40.2%]	**<0.001**
Male, *n* (%)	231 (58.5%) [67%]	47 (50%) [52%]	184 (61.7%) [71.2%]	**0.044**
Arterial hypertension, *n* (%)	246 (62.3%) [60%]	73 (77.7%) [74.5%]	173 (57.5%) [55.9%]	**<0.001**
Diabetes mellitus, *n* (%)	117 (29.6%) [24.8%]	31 (33%) [21.6%]	86 (28.7%) [25.7%]	0.425
Dyslipidemia, *n* (%)	186 (47.1%) [41.5%]	52 (55.3%) [47.1%]	134 (44.7%) [39.9%]	0.071
Ischemic heart disease, *n* (%)	44 (11.2%) [7.2%]	18 (19.1%) [6.9%]	26 (8.7%) [7.3%]	**0.005**
Chronic renal failure, *n* (%)	38 (9.6%) [8.9%]	11 (11.7%) [13.7%]	27 (9%) [7.6%]	0.433
Bronchopathy, *n* (%)	50 (12.7%) [16.7%]	19 (20.2%) [29.4%]	31 (10.3%) [13.1%]	**0.012**
Thyroid disease, *n* (%)	35 (8.9%) [7.2%]	9 (9.6%) [13.7%]	26 (8.6%) [5.3%]	0.780
Previous ischemic stroke, *n* (%)	57 (14.6%) [13.5%]	16 (17.2%) [19.6%]	41 (13.8%) [11.7%]	0.411
NIHSS score on admission, median (IQR)	3 (6) [4]	5.5 (11.5) [7]	3 (6) [3]	**<0.001**

*Laboratory measures*				
NT-ProBNP levels, median (IQR), pg/mL	214 (679) [186]	735 (1661) [784]	156 (436) [125]	**<0.001**
NT-ProBNP ≥250 pg/mL, *n* (%)	188 (47.6%) [41.7%]	71 (75.5%) [79.4%]	117 (38.9%) [31%]	**<0.001**
*Echocardiographic features*				
Left atrial enlargement, *n* (%)	159 (40.3%) [27.4%]	67 (71.3%) [59.8%]	92 (30.6%) [18.2%]	**<0.001**
Left ventricular hypertrophy, *n* (%)	157 (40.3%) [39.3%]	47 (50.5%) [36.3%]	110 (37%) [40.2%]	**0.021**
Diastolic dysfunction, *n* (%)	89 (23%) [36%]	17 (18.7%) [34.3%]	72 (24.3%) [36.5%]	0.263
*Neuroimaging features*				
Cortical topography of stroke, *n* (%)	223 (56.5%) [60%]	73 (77.7%) [81.4%]	150 (49.8%) [53.9%]	**<0.001**
Intracranial large vessel occlusion, *n* (%)	124 (31.4%) [32%]	48 (51.1%) [51%]	76 (25.2%) [26.5%]	**<0.001**
Chronic cortical stroke, *n* (%)	92 (23.3%) [8.9%]	29 (30.9%) [17.6%]	63 (20.9%) [6.4%]	**0.047**

*AF assessment*				
Median telemetry duration (IQR), days	2 (2) [4]	3 (2) [4]	2 (2) [4]	0.468
Median time until diagnosis (IQR), days	3 (13.5) [2]	3 (13.5) [2]	N.A.	N.A.
24 h Holter monitoring, *n* (%)	91 (23%) [17.2%]	15 (16%) [16.7%]	76 (25.2%) [17.3%]	0.062
28 days Holter monitoring, *n* (%)	70 (17.7%) [17.2%]	24 (25.5%) [18.6%]	46 (15.3%) [16.8%]	**0.023**

IQR: interquartile range; N.A.: not applicable; NT-ProBNP: N-terminal pro-B-type natriuretic peptide. ^∗^Brackets indicate absolute frequency or median, as appropriate. ^∗∗^Comparisons between the atrial fibrillation (AF) and non-AF groups of the external validation cohort. Significant values are highlighted in bold.

## Data Availability

The original contributions presented in the study are included in the article; further inquiries can be directed to the corresponding author.

## Supplementary Materials

Supplementary Materials In the Supplementary material, there is a gender analysis, with Table S1 showing the baseline characteristics of the patients by gender and Figure S1 showing the ROC curves by gender in the external cohort and the comparison of both AUCs.
