# Supplementary material for: External Validation of SAFE Score to Predict Atrial Fibrillation Diagnosis after Ischemic Stroke: A Retrospective Multicenter Study
Source: Stroke Res Treat. 2023 Dec 7;2023:6655772. doi: 10.1155/2023/6655772 (PMC10721350; doi:10.1155/2023/6655772)

**SUPPLEMENTARY MATERIAL**

**Table S1.** Patient characteristics by gender

* 3 missing values

| Variable | All patients  (*n* = 395) | Male patients  (*n* = 231) * | Female patients  (*n* = 161) * | *P*-value |
| --- | --- | --- | --- | --- |
| Clinical variables |  |  |  |  |
| Median age (IQR), years | 72 (20) | 70 (18) | 75 (18) | **<0.001** |
| Age ≥ 65 years, n (%) | 269 (68.1%) | 147 (63.6%) | 121 (75.2%) | **0.016** |
| Arterial hypertension, n (%) | 246 (62.3%) | 138 (59.7%) | 108 (67.1%) | 0.139 |
| Diabetes mellitus, n (%) | 117 (29.6%) | 74 (32%) | 42 (26.3%) | 0.218 |
| Dyslipidemia, n (%) | 186 (47.1%) | 104 (45%) | 81 (50.6%) | 0.275 |
| Ischemic heart disease, n (%) | 44 (11.2%) | 31 (13.5%) | 13 (8.2%) | 0.254 |
| Chronic renal failure, n (%) | 38 (9.6%) | 27 (11.7%) | 11 (6.8%) | 0.110 |
| Bronchopathy, n (%) | 50 (12.7%) | 37 (16%) | 13 (8.2%) | **0.02** |
| Thyroid disease, n (%) | 35 (8.9%) | 13 (5.6%) | 22 (13.7%) | **0.006** |
| Previous ischemic stroke, n (%) | 57 (14.6%) | 35 (15.4%) | 22 (13.7%) | 0.661 |
| NIHSS score on admission, median (IQR) | 3 (6) | 3 (7) | 4 (7) | 0.292 |
| Laboratory measures |  |  |  |  |
| NT-ProBNP levels, median (IQR), pg/mL | 214 (679) | 169 (539) | 297 (880) | **0.004** |
| NT-ProBNP ≥ 250 pg/mL, n (%) | 188 (47.6%) | 97 (42%) | 90 (55.9%) | **0.006** |
| Echocardiographic features |  |  |  |  |
| Left atrial enlargement, n (%) | 159 (40.3%) | 94 (40.7%) | 64 (39.8%) | 0.852 |
| Neuroimaging features |  |  |  |  |
| Cortical topography of stroke, n (%) | 223 (56.5%) | 131 (56.7%) | 91 (56.5%) | 0.971 |
| Intracranial large vessel occlusion, n (%) | 124 (31.4%) | 62 (26.8%) | 61 (37.9%) | **0.02** |
| Chronic cortical stroke, n (%) | 92 (23.3%) | 52 (22.5%) | 39 (24.2%) | 0.692 |
| AF assessment |  |  |  |  |
| AF diagnosis | 94 (23.8%) | 47 (20.3%) | 47 (29.2%) | **0.044** |
| Median telemetry duration (IQR), days | 2 (2) | 2 (2) | 2 (2) | 0.669 |

**Figure S1.** ROC curves by gender


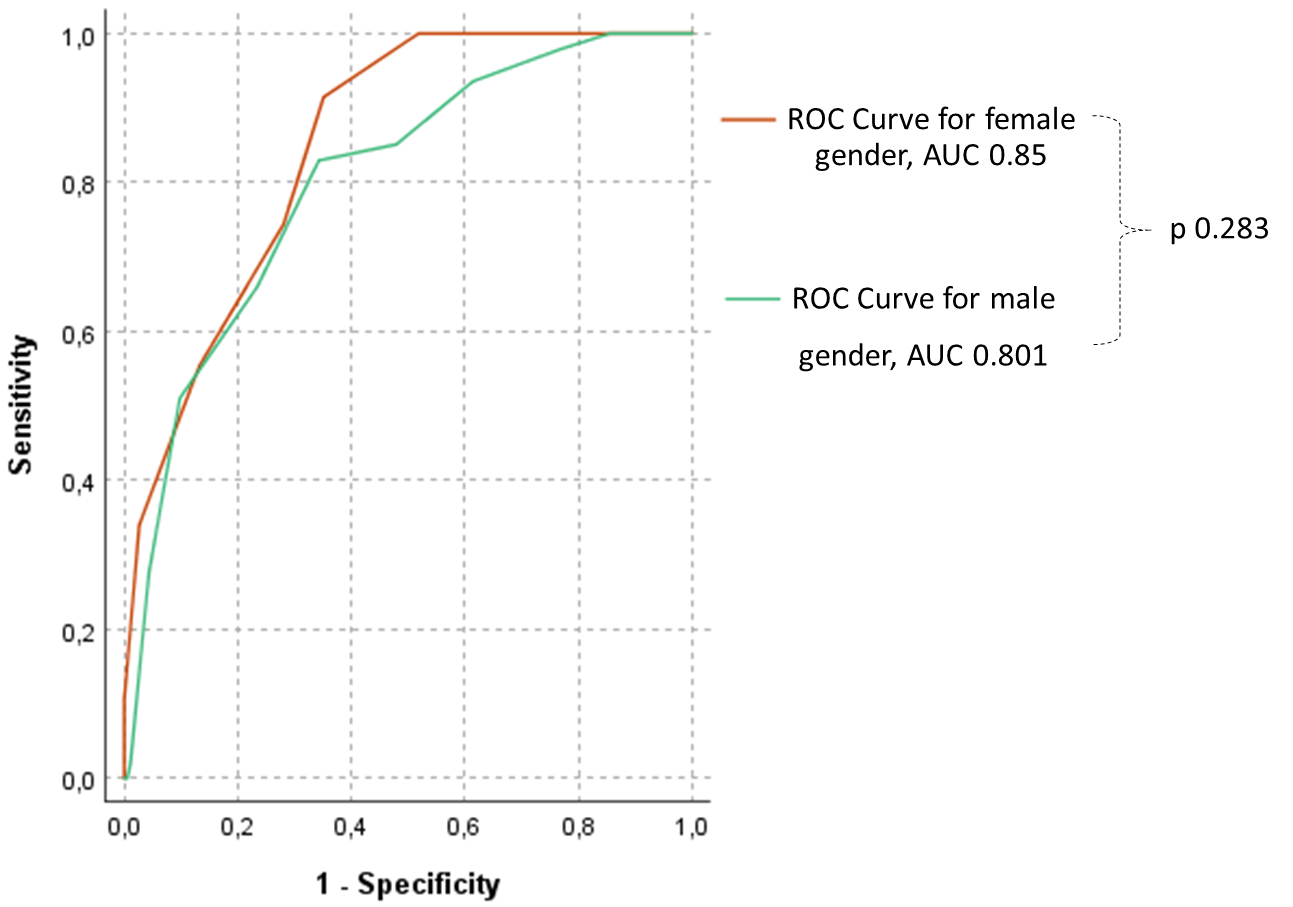

Supplement: Supplementary Materials — In the Supplementary material, there is a gender analysis, with Table S1 showing the baseline characteristics of the patients by gender and Figure S1 showing the ROC curves by gender in the external cohort and the comparison of both AUCs. [file 6655772.f1.zip › SUPPLEMENTARY MATERIAL.docx]
